# Hippocampus Parcellation via Discriminative Embedded Clustering of fMRI Functional Connectivity

**DOI:** 10.3390/brainsci13050757

**Published:** 2023-05-03

**Authors:** Limin Peng, Chenping Hou, Jianpo Su, Hui Shen, Lubin Wang, Dewen Hu, Ling-Li Zeng

**Affiliations:** 1 College of Intelligence Science and Technology, National University of Defense Technology, Changsha 410073, China; 2 College of Liberal Arts and Science, National University of Defense Technology, Changsha 410073, China; 3 The Brain Science Center, Beijing Institute of Basic Medical Sciences, Beijing 102206, China

**Keywords:** discriminative embedded clustering, hippocampus, functional connectivity, parcellation, spatial navigation

## Abstract

Dividing a pre-defined brain region into several heterogenous subregions is crucial for understanding its functional segregation and integration. Due to the high dimensionality of brain functional features, clustering is often postponed until dimensionality reduction in traditional parcellation frameworks occurs. However, under such stepwise parcellation, it is very easy to fall into the dilemma of local optimum since dimensionality reduction could not take into account the requirement of clustering. In this study, we developed a new parcellation framework based on the discriminative embedded clustering (DEC), combining subspace learning and clustering in a common procedure with alternative minimization adopted to approach global optimum. We tested the proposed framework in functional connectivity-based parcellation of the hippocampus. The hippocampus was parcellated into three spatial coherent subregions along the anteroventral–posterodorsal axis; the three subregions exhibited distinct functional connectivity changes in taxi drivers relative to non-driver controls. Moreover, compared with traditional stepwise methods, the proposed DEC-based framework demonstrated higher parcellation consistency across different scans within individuals. The study proposed a new brain parcellation framework with joint dimensionality reduction and clustering; the findings might shed new light on the functional plasticity of hippocampal subregions related to long-term navigation experience.

## 1. Introduction

In recent years, resting-state functional magnetic resonance imaging (fMRI) has emerged as a powerful tool in identifying the locations of putative brain functional borders, predicting individual phenotypes, decoding the brain task activation and diagnosing clinical mental diseases [1,2,3]. The parcellation of resting-state fMRI is testified to be crucial for understanding the functional segregation and integration properties of the brain organization [4,5], in which a pre-defined brain region can be subdivided into several spatially coherent subregions with homogeneous functional patterns. In fact, the parcellation can be regarded as an unsupervised clustering task, in which the voxels of a region are allocated into different clusters with high intra-cluster similarity and inter-cluster differences. 

In previous studies, resting-state functional connectivity was shown to have advantages as the clustering feature in identifying specialized functional subregions [6,7,8,9,10]. Moreover, functional connectivity is believed to have natural cluster structure considering the existence of hubs in it [11]; this assumption is also the premise of parcellation. It should be noted that the connectivity features, in many situations, have very high dimensions, often far exceeding the number of voxels, which brings huge challenges to parcellation tasks. One direct way to tackle these challenges is to first employ dimensionality reduction approaches, such as principal component analysis (PCA), and then cluster the embedded features in low-dimensional space. However, this method is quite easy to trap into local optimum, as these two techniques are conducted separately. The procedure of dimensionality reduction does not take the requirement of clustering into account. We believe that the performance of clustering will be improved if the two techniques are performed simultaneously.

To avoid the local optimum problem possibly confronted in the brain parcellation, we proposed a novel algorithm referred to as discriminative embedded clustering (DEC), and confirmed its superiority in the clustering of high-dimensional data. In DEC, there are two issues to be addressed, the first being dimensionality reduction and the second being clustering. When we fix one group parameter, the subproblem is joint convex to the variables; thus, alternating minimization can be used to obtain the global optimum [12]. Numerous simulation results on several benchmark datasets have confirmed that the DEC outperformed related state-of-the-art clustering approaches, as well as existing joint dimensionality reduction and clustering methods.

Based on the DEC, we also introduced a new brain parcellation framework, and attempted to employ it in the functional parcellation of hippocampus. The importance of hippocampus in memory and spatial navigation for human beings is well established [13,14,15]. Numerous studies have found that the hippocampus is a functionally heterogeneous region that can be parcellated into three dissimilar compartments along the anterior–posterior or ventral–dorsal axis [16,17,18,19,20,21]: the dorsal hippocampus, ventral hippocampus and the intermediate hippocampus. These subdivisions are involved in different functions and project with differing neural pathways. In specific, the dorsal hippocampus is reported to participate in many primarily cognitive functions, including spatial memory and navigation. The ventral hippocampus is reported to serve for self-emotion and affective process [19,22], and the intermediate hippocampus has overlapping attributes with both the ventral and dorsal part [19]. However, the functional connectivity maps of hippocampal subregions, and the relationships between subregions and spatial navigation, remain elusive. 

Previous studies have reported that long-term navigation experience would change the gray matter volumes of hippocampus, indicating the structural plasticity of human brains [23,24]. Furthermore, in our recent study, significant differences in functional connectivity between taxi drivers and non-driver controls were observed in entorhinal cortex, reflecting the potential influence of extensive navigation training on the functional organization of healthy human brains [25]. Since entorhinal cortex is the main interface conveying the object- and scene-related information to the hippocampus, we intend to explore the possibility that the effect of long-term navigation experience can extend from the entorhinal cortex to the adjacent hippocampus.

We have two specific goals in this study. Firstly, we aimed to test the availability of our DEC-based parcellation framework; thus, we subdivided the hippocampus into three functional subregions, and examined the differences of functional connectivity maps among these subregions. Secondly, choosing the resulting three hippocampal subregions as our regions of interest (ROIs), we aimed to explore the navigation-related functional connectivity changes of hippocampus. For each ROI, we compared the functional connectivity maps of 20 taxi drivers with those of 20 control subjects with no driving experience, which helped to advance our understanding of how the brain supports navigation.

## 2. Materials and Methods

### 2.1. Participants and Data Acquisition

The fMRI data used for hippocampal parcellation comprised 2 resting-state scans from 20 healthy subjects (10 males, 10 females, age: 22 to 36 years), selected from the Human Connectome Project (HCP, http://www.humanconnectomeproject.org/, accessed on 1 May 2020). Participants were scanned on a customized 3T Siemens MRI scanner with their eyes open, keeping still and conspicuous. Images were acquired with the following parameters: TR = 0.72 s, TE = 33.1 ms, flip angle = 52 degrees, bandwidth = 2290 Hz/Px, field of view (FOV) = 208 × 180 mm^2^, number of slices = 72, voxel size =2.0 × 2.0 × 2.0 mm^3^, and number of time points = 1200.

Comparison analysis was performed on 20 licensed taxi drivers and 20 healthy control subjects (right-handed) with no driving experience, and the two groups were matched for sex, age, and educational level. All of the taxi drivers, who worked approximately 8 h a day, had been driving for an average time of 11.6 years (range 6–23 years), and the average time period spent working as licensed taxi drivers was 4.9 years (range 1–14 years). The participants in the control group did not know how to drive, and their transportation modes consisted of walking and the bus system. Detailed demographic information of the included subjects was provided in Table 1. None of them had a history of major head trauma, neurological disorder, or addiction to alcohol or drugs. The participants were all recruited in Chongqing, China, which is located in a mountainous region with a complicated traffic situation. The Institutional Review Board of Southwest University approved the study, and all subjects provided written informed consent in order to participate. The resting-state fMRI data were collected using a Siemens Trio 3-T MRI scanner in the Key Laboratory of Cognition and Personality (Southwest University), Ministry of Education, China. The imaging parameters were as follows: number of axial slices = 32, TR = 2000 ms, TE = 30 ms, slice thickness = 3.0 mm, flip angle = 90°, and FOV = 200 × 200 mm^2^. For each subject, the resting-state scan lasted 8 min, and 240 volumes were obtained.

### 2.2. Data Preprocessing

The fMRI data used for hippocampal parcellation were, firstly, pre-processed with the standard HCP minimal pre-processing pipeline [26,27], including rigid-body motion correction, cross-modal registration, MNI spatial transformation, denoising with the ICA-based “fix” algorithm and regressing out nuisance signals. Moreover, spatial smoothing with a Gaussian filter of 4 mm full-width half-maximum (FWHM) kernel and band-pass filtering (0.01–0.08 Hz) were further applied. After these steps, the 2 scans of resting-state were temporally concatenated for each subject, and the resulting time series could be utilized for the subsequent clustering analysis.

SPM8 software (Welcome Department of Imaging Neuroscience, University College London, UK, http://www.fil.ion.ucl.ac.uk/spm/, accessed on 1 April 2018) were applied for the pre-processing of fMRI data used for comparison analysis. In specific, the fMRI data of taxi drivers and normal controls were pre-processed using previously described procedures [28,29,30]. The first 10 volumes of each scan were discarded to avoid the magnetic saturation effects. The slice timing and head motion correction were then performed on the remaining fMRI images. All participants in this study had less than 2 mm translation and 2° of rotation in any of the *x*, *y*, and *z* axis. Next, the volumes were normalized (3 mm isotropic voxels) to the standard EPI template in the Montreal Neurological Institute (MNI) space. Subsequently, the resulting images were spatially smoothed (FWHM = 6 mm) and temporally filtered with a Chebyshev band-pass filter (0.01–0.08 Hz). Finally, to further reduce the signal noise, the filtered data were regressed with the six head motion parameters and the white matter (WM), cerebrospinal fluid (CSF) and whole brain mean signals, as well as their first-order derivative terms. The residuals of the regression were used for further analysis.

### 2.3. Discriminative Embedded Clustering

In this study, we introduced a novel functional parcellation framework based on the DEC algorithm, and attempted to employ it in the hippocampal subdivision. As a novel algorithm for high dimensional data clustering, DEC combines subspace learning and clustering in a unified framework. Different from traditional approaches, which conduct dimensionality reduction and clustering in sequence, DEC performs the two procedures simultaneously. As known, the objective function of PCA is:

(1) $$\max Tr(YY^{T})=\max Tr(Q^{T}XX^{T}Q)=\max Tr(Q^{T}S_{t}Q)$$

where $X=[x_{1},\cdots x_{n}]\in R^{D\times n}$ is the data matrix in original space, $Y=[y_{1},\ldots ,y_{n}]\in R^{d\times n}$ is the data matrix in low dimensional subspace, $Q\in R^{D\times d}$ is the transformation matrix and $S_{t}$ is the variance matrix.

The objective function of K-means is:

(2) $$\min\;{\left\|Y-GF^{T}\right\|}_{F}^{2}$$

where $G\in R^{d\times c}$ is the cluster centroid matrix, and $F=[f_{1},\ldots ,f_{n}]\in R^{n\times c}$ is the cluster indicator matrix. Inspired by thinking on how to unify PCA and K-means, DEC unified subspace learning and clustering through sharing the transformation matrix Q. By adding the constraint $Y=Q^{T}X$ and a balance parameter λ, we can identify the formulation of DEC:

(3) $$\arg\underset{Q,G,F}{\max}\;\;\;Tr(Q^{T}S_{t}Q)-\lambda {\left\|Q^{T}X-GF^{T}\right\|}_{F}^{2}\;s.t.\;Q^{T}Q=I$$

We can identify different methods within the general framework through choosing suitable parameter λ, which balances the effects between dimensionality reduction and clustering in subspace. For example, DEC is equivalent to performing PCA and K-means in sequence when λ towards zero. When λ=1, DEC is equivalent to conducting OCM (Orthogonal Centroid Method) and K-means alternately.

Since (3) is not joint convex with respect to Q, G and F, alternating minimization is proposed to find its global optimal solution [12]. Overall, when the number of clusters is C, the dimensionality of the embedding subspace d and the balance parameter λ were given; the procedure of DEC is described in Algorithm 1.
**Algorithm 1** DEC method**Input:**$\;\mathrm{Dataset}:\;X=[x_{1},\cdots x_{n}]$, number of clusters C, dimensionality of the embedding subspace d, and balance parameter λ. **Output:** Transformation matrix Q, cluster centroid matrix G and cluster indicator F.**1.** Initialize Q by performing PCA, and initialize F by conducting K-means on $Q^{T}X$. **2.** Optimize F by fixing Q and G. Similarly, optimize Q and G by fixing F. To avoid local optimization, initialize F several times randomly and update F according to relevant rules. **3.** Repeat Step 2 until convergence.

### 2.4. Functional Parcellation of the Hippocampus

**Figure 1:**
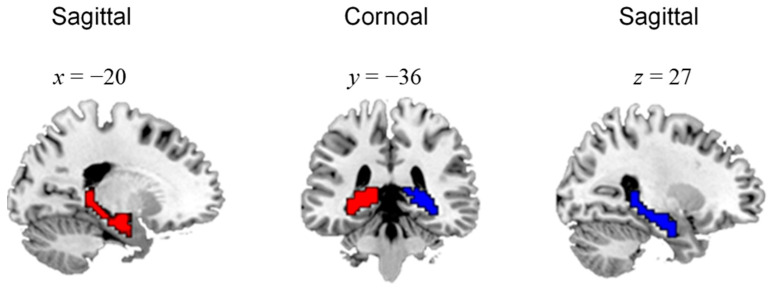
Mask of hippocampus shown in sagittal and coronal view.

In this study, based on the pre-processed fMRI data, our DEC-based parcellation framework was employed to divide the hippocampus into three functional subregions. Firstly, a uniform mask of the hippocampus was obtained in MNI space using the software WFU Pick Atlas (http://fmri.wfubmc.edu/software/PickAtlas/, accessed on 10 April 2022) (Figure 1). Secondly, for each subject, the functional connectivity matrix was calculated for the hippocampus parcellation. Specifically, we used 114 cortical regions as the target ROIs [31], and calculated the Pearson’s correlation coefficients between the time series of voxels in the hippocampus mask and those of 114 ROIs. The resulting correlation matrix was then *z*-transformed to improve normality. Finally, the individual *z*-transformed functional connectivity matrix was input into the DEC approach, which output the clustering label for each hippocampal voxel. The clustering was performed for the left and right hippocampus, respectively, and the number of clusters was set at 3 in order to parcellate the hippocampus into three functional subregions. After clustering, the parcellation results for each subject were obtained.

It should be noted that there are two important pre-determined parameters in DEC, which are the dimensionality of the embedding subspace d and the balance parameter λ. We can use grid search to determine the optimal parameters. Here, the examined dimensionality d varied from 2 to 40, with a step size of 2, and the balance parameter λ varied from 0 to 40, with a step size of 2. The average entropy and the silhouette width (SI) were separately chosen to evaluate the external and internal clustering validation [32,33].

To estimate the stability of the cluster solutions, 20 half-length scans were generated randomly from the whole scan for each subject. To ensure the intra-subject stability of the connectivity matrices calculated from these scans, we performed a leave-one-out stability analysis. Specifically, we averaged the connectivity matrices for 19 scans, and calculated the correlation between this averaged connectivity pattern and the connectivity pattern of the left-out scan for each voxel. This process was repeated 20 times, each time with a different scan as the left-out scan. The voxel-wise correlations were then averaged to obtain the average stability diagram. Accordingly, the division was stable among these 20 scans for each subject, and the consistency of clustering across scans could be used to evaluate the performance of the algorithms. Let $L_{S}=[l_{s}(1),l_{s}(2),\ldots ,l_{s}(N)]$ denote the label matrix for subjects, and $l_{s}(i)\in \left\{1,2,3\right\}$. The probability of voxel $v_{i}$ being classified as subdivision c is given by

(4) $$\mathrm{Pr}(v_{i}=c)=\frac{\sum_{sc}\delta (l_{s}(i),c)}{SC}$$

where δ(.,.) is the Kronecker delta function, and SC is the number of scans (SC = 20 here). The uncertainty of label assignment at a single voxel can be assessed via the discrete entropy,

(5) $$H(i)=-\sum_{c=1}^{3}\mathrm{Pr}(v_{i}=c)\log(\mathrm{Pr}(v_{i}=c))$$

The intra-subject cluster consistency is defined based on the average entropy over all voxels.

(6) $$H=\frac{1}{N}\sum_{v_{i}}H(i)$$

SI was chosen to quantify the functional homogeneity of segmented subregions. Here, SI was defined in terms of Euclidean distance and calculated via the following formulation:

(7) $$SI=\frac{1}{C}\sum_{i=1}^{C}\frac{b_{i}-a_{i}}{\max\{a_{i},b_{i}\}}$$

$a_{i}$—the average distance between every pair of voxels assigned to the *i*-th cluster.
$b_{i}$—the average distance between within and out of the *i*-th cluster.
C—the number of clusters.

**Figure 2:**
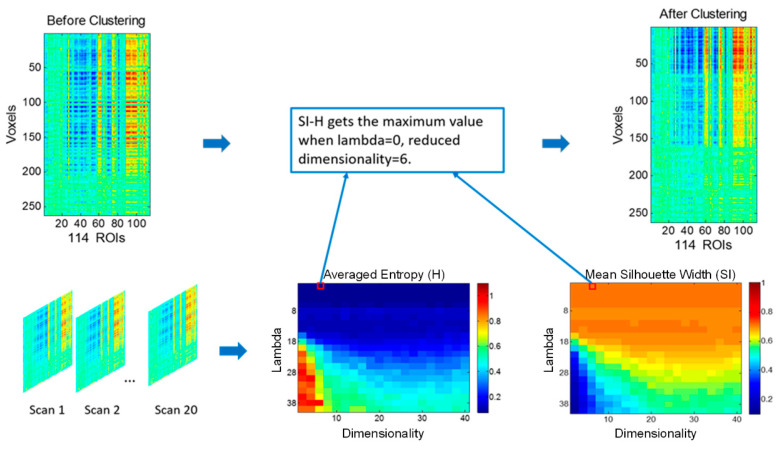
Procedure of determining optimal parameters d and λ in DEC.

An optimal partitioning of DEC with respect to d and λ was determined when SI−H reached the maximum value (Figure 2). 

For each subject, we obtained the cluster labels of all voxels in hippocampus (L/R), and further obtained the hippocampal subregions individually. The individual correlation maps were then created through calculating the Pearson’s correlation coefficients between the mean time series of each subregion and those of each voxel in the whole brain for each subject. These correlation maps were *z*-transformed to improve normality. Subsequently, a voxel-wise one-sample *t*-test was conducted on these *z*-value maps to determine the brain regions significantly correlated with each hippocampal subregion, respectively. The significant level was set at *p* < 0.05, with a false discovery rate (FDR) corrected with cluster size >10 voxels. Finally, we aimed to examine statistical differences among the functional connectivity patterns of these three subregions. Specifically, paired-samples *t*-tests (*p* < 0.05, Bonferroni corrected) were conducted on the functional connectivity of ipsilateral hippocampal subregions with 114 target ROIs, which could be further divided into six functional networks, including the visual network, somatomotor network, dorsal–attention network, ventral attention network, default network, and control network [31]. 

### 2.5. Comparison Analysis

To further investigate the differences of resting-state hippocampal functional connectivity between the taxi drivers and non-driver controls, a ROI-based comparison analysis was conducted. After obtaining the parcellation result for each individual, we used a standard majority-voting scheme to identify the hippocampal subregions on the group level. For each voxel in the hippocampus, we calculated its probability for the three clusters across 20 HCP subjects, and assigned this voxel into the cluster with the highest value of probability to find the group-level template of hippocampal subdivision. In each subdivision, we then chose the regions, within which the voxels all had a value of probability exceeding 0.6, as our ROIs. Next, in taxi drivers and controls, we averaged the time courses of voxels in each ROI to obtain the signals of the corresponding subregion, and obtained its functional connectivity map through calculating the Pearson’s correlation coefficients between the signals of each subregion and that of each voxel in the whole brain. Fisher’s *z* transformation was applied to these maps, and one-sample *t*-tests (*p* < 0.05, FDR corrected) were conducted on the *z*-maps of drivers and controls, respectively, to identify the regions with significant connectivity to the corresponding hippocampal subregion. We combined the spatial maps of the two groups to obtain the connectivity mask for each ROI, and performed voxel-wise two-sample *t*-tests (*p* < 0.05, AlphaSim corrected) within this mask to determine the group differences. The AlphaSim correction (cluster radius connection: rmm = 7; number of Monte Carlo simulations = 1000) was performed using the AlphaSim program in REST toolbox (http://www.restfmri.net/, accessed on 10 March 2022), which applied Monte Carlo simulation to calculate the probability of false positive detection by taking into consideration both the individual voxel probability threshold and cluster size [34]. 

## 3. Results

### 3.1. Functional Connectivity-Based Parcellation of the Hippocampus

**Figure 3:**
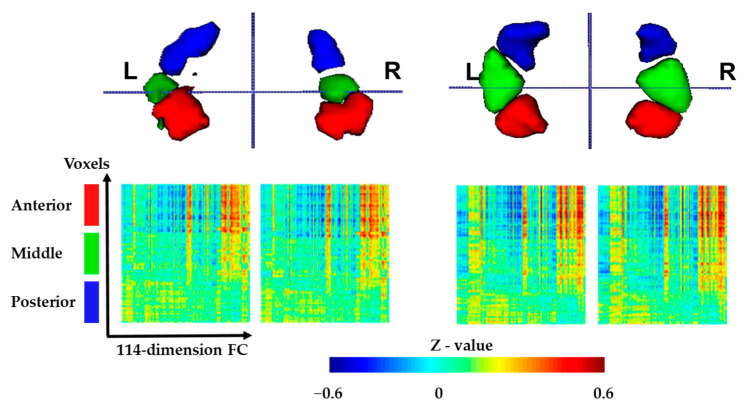
Functional connectivity-based parcellation of bilateral hippocampus on two sample subjects. Top row shows the three clusters mapped on brain. Bottom row shows the reordered functional connectivity matrices of left and right hippocampus for different subjects. Anterior = anterior hippocampus; middle = middle hippocampus; posterior = posterior hippocampus.

For each subject, we generated the individual parcellation of the hippocampus into three subregions in the standard MNI space. Figure 3 shows the clustering results of two sample subjects, with voxels assigned to distinct clusters displayed in red, green and blue, respectively. After mapping each voxel in the bilateral hippocampus onto the brain mask, an anteroventral–posterodorsal spatial distribution of hippocampal subdivisions was obtained. The reordered functional connectivity matrices of the bilateral hippocampus for different subjects are also shown in Figure 3. It is observed that voxels within the same cluster were functionally similar, whereas voxels between clusters showed different connectivity patterns.

**Figure 4:**
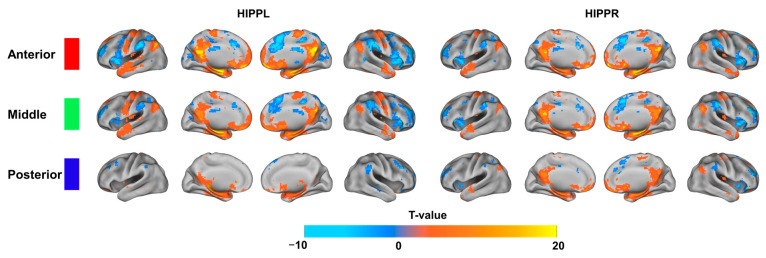
Functional connectivity patterns of each cluster from 20 subjects (One-sample *t*-tests, FDR, *p* < 0.05, cluster size > 10). HIPPL = left hippocampus; HIPPR = right hippocampus.

Considering individual variation, our hypothesis stated that the resulting subregions were functionally consistent across all subjects, although the spatial distribution of the three subregions might differ. For each subject, we selected the three subregions as seeds to calculate the functional connectivity with all the voxels in the brain, resulting in functional connectivity maps of the three subregions. Group-level analyses (voxel-wise one-sample *t*-tests, *p* < 0.05, FDR corrected) were then performed on these maps to examine the connectivity patterns of the three parcellated clusters. Figure 4 shows the functional connectivity patterns of each subregion in the bilateral hippocampus across 20 subjects. We observed that the subregion located in the anterior part of the hippocampus (color coded in red) was positively correlated with the orbitofrontal cortex (OFC), anterior cingulate cortex (ACC), precentral gyrus, precuneus and some lateral and medial temporal regions, but negatively correlated with the superior and middle frontal gyrus, insula, cingulate gyrus, inferior parietal lobule and middle temporal gyrus. The posterior part of the hippocampus (color coded in blue) showed relatively positive correlation with the parahippocampal gyrus, posterior cingulate cortex (PCC) and precuneus, as well as some occipital regions, and the regions negatively correlated with it were mainly located in the lateral and medial frontal lobe.

**Figure 5:**
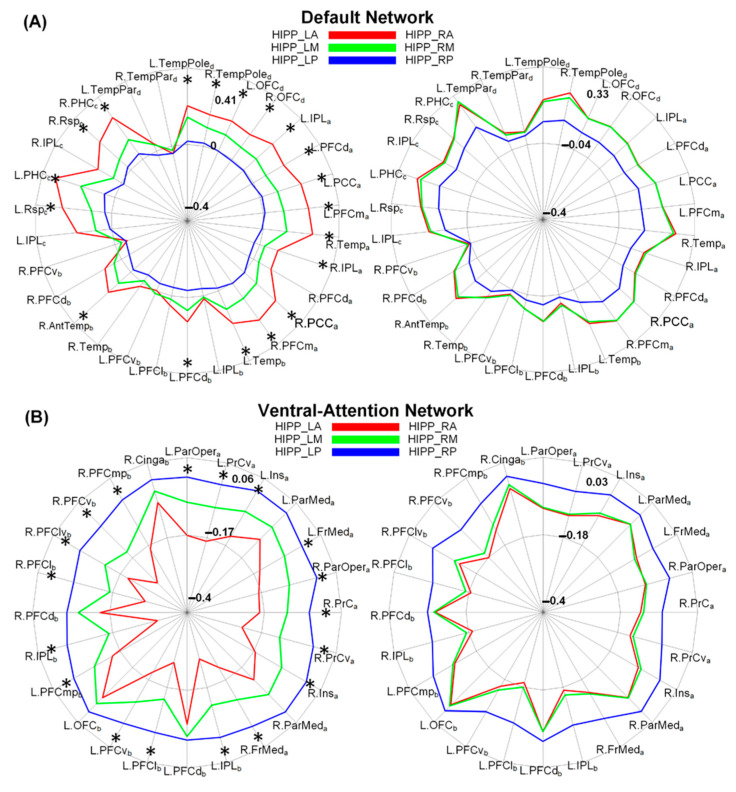
Radar plots demonstrated functional connectivity of hippocampal subregions with default network (**A**) and ventral–attention network (**B**), which were defined by [31]. Red, green and blue lines, respectively, denote functional connectivity of anterior, middle and posterior hippocampal subregion with six networks. Paired *t*-tests were conducted to identify significant differences of functional connectivity among three subregions on network level (* *p* < 0.05, Bonferroni corrected). HIPP_LA = left anterior hippocampus; HIPP_LM = left middle hippocampus; HIPP_LP = left posterior hippocampus; HIPP_RA = right anterior hippocampus; HIPP_RM = right middle hippocampus; HIPP_RP = right posterior hippocampus.

In addition, based on the functional connectivity with the 114 ROIs, we further compared the connectivity differences of the hippocampal subregions on the network levels. As seen in Figure 5, for the left hippocampus, significant differences of functional connectivity among the three subregions were only located in the default network and the ventral-attention network (*p* < 0.05, Bonferroni corrected). Specifically, the left anterior subregion exhibited the most positive connectivity with the default network, as well as the most negative connectivity with the ventral attention network, followed by the middle subregion and the posterior subregion. However, similar network-level differences of functional connectivity were not observed for the right hippocampus.

### 3.2. Performance of DEC-Based Parcellation Framework Compared with Existing Methods

**Figure 6:**
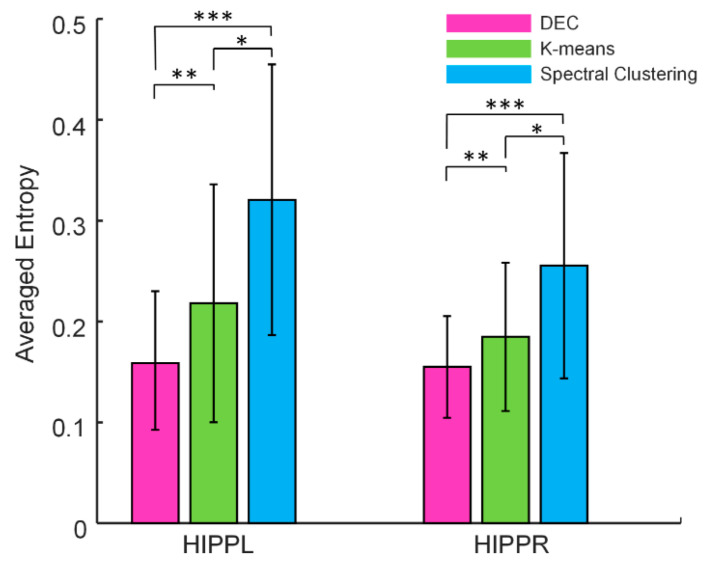
Comparison of partitioning on average entropy for 20 subjects between different clustering algorithms, including K-means, spectral clustering and DEC. Paired-samples *t*-tests: * *p* < 0.05; ** *p* < 0.005; *** *p* < 0.001. HIPPL = left hippocampus; HIPPR = right hippocampus.

We compared our DEC-based parcellation framework with other traditional parcellation algorithms, including K-means and spectral clustering. In practice, the average entropy was used to evaluate the stability of parcellation, and a better framework should achieve more consistent cross-scan parcellation results within individuals. As mentioned above, for the 20 different subjects, 20 values of average entropy were obtained to evaluate the clustering consistency of each individual, respectively. By averaging the 20 values, Figure 6 demonstrates the comparison of the parcellation results on average entropy between different clustering algorithms, including K-means, spectral clustering and DEC. As can be seen, the DEC has the smallest value of averaged entropy (paired-samples *t*-test, *p* < 0.005), followed by K-means and spectral clustering, which indicates that our framework is better at catching the shared components of intra-individual fMRI scans than existing methods.

### 3.3. Discriminative Functional Patterns of the Hippocampal Subregions in the Taxi Drivers 

**Figure 7:**
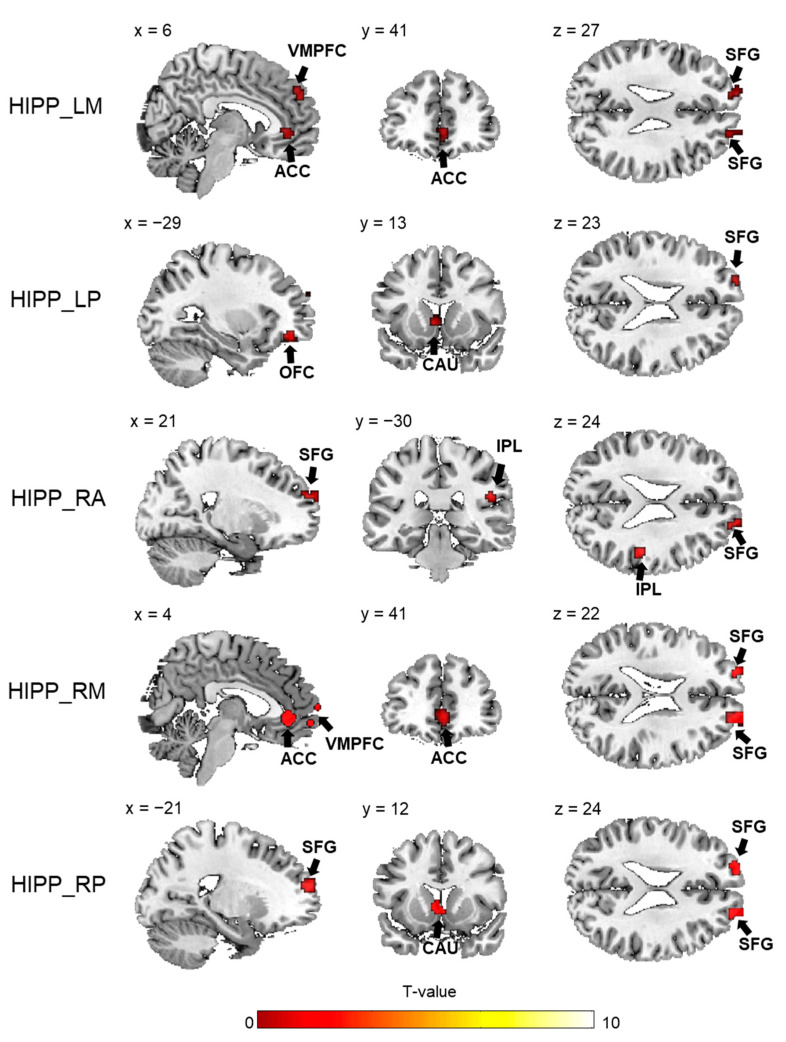
Comparison of hippocampal connectivity patterns between taxi drivers and control subjects with no driving experience. Two-sample *t*-test results (*p* < 0.001, uncorrected) are mapped separately for right anterior hippocampal subregions, bilateral middle subregions and bilateral posterior subregions. HIPP_LA = left anterior hippocampus; HIPP_LM = left middle hippocampus; HIPP_LP = left posterior hippocampus; HIPP_RA = right anterior hippocampus; HIPP_RM = right middle hippocampus; HIPP_RP = right posterior hippocampus.

As shown in Figure 7 and Table 2, statistical functional connectivity differences were observed between taxi drivers and non-driver controls (two-sample *t*-tests, *p* < 0.05, AlphaSim corrected). For taxi drivers, functional connectivity was significantly diminished between several regions and hippocampal subdivisions compared with controls. Specifically, both the left and right middle parts showed reduced functional connectivity with the bilateral ACC, superior frontal gyrus and right ventral medial prefrontal cortex (VMPFC), in addition to the reduced functional connectivity between the right middle part and left superior temporal gyrus. For the posterior hippocampal subdivisions, both the left and right parts exhibited reduced functional connectivity with the left superior frontal gyrus and caudate nucleus. As for the anterior subregions, only the right anterior part showed reduced functional connectivity with the right superior frontal gyrus and inferior parietal lobule. No increased hippocampal functional connectivity was found in taxi drivers compared with controls.

## 4. Discussion

Traditional brain parcellation methods tend to perform dimensionality reduction and clustering separately, which may easily resolve the problem of local optimum. To tackle this problem, in this study, we proposed a novel brain parcellation framework based on the DEC method, and attempted to employ it in the functional parcellation of human hippocampus. The hippocampus was subdivided into three spatial coherent subregions along its anteroventral-posterodorsal axis; the voxels in the same cluster exhibited similar connectivity patterns, while the voxels among different clusters showed distinct connectivity patterns. Furthermore, by calculating the average entropy of the parcellation results with differing cluster methods, we found that the results of DEC demonstrated the highest consistency across different fMRI scans compared with K-means and spectral clustering. In addition, one-sample *t*-tests were conducted to identify the areas showing significant functional connectivity with each hippocampal subregion. We found that the anterior part of the hippocampus had significant positive functional connectivity with the orbitofrontal cortex—ACC—and precuneus, but negative connectivity with the insular and inferior parietal lobule. Meanwhile, the posterior part of the hippocampus showed significant positive correlation with the parahippocampal gyrus and PCC. Moreover, by contrasting the hippocampal functional connectivity maps between taxi drivers and normal controls, statistical differences of the hippocampal functional connectivity were observed between the two groups. The results exhibited the advantage of our parcellation framework in controlling intra-subject clustering stability, and demonstrated the navigation-related changes of the hippocampal functional maps in taxi drivers, which might shed new light on the analysis of hippocampal functional patterns. 

Through the framework, we subdivided the hippocampus into three functionally heterogeneous subregions based on the functional connectivity profiles. Notably, the results obtained using the DEC framework showed the lowest degree of inter-scan discrepancy compared to other widely-used clustering algorithms, such as K-means and spectral clustering. This result indicates that our method performs better at controlling intra-subject fluctuation of neural signals. As known, in K-means algorithm, it is supposed that the samples have a hyperspherical or Gaussian distribution in the high-dimension feature space, and that these samples are located symmetrically around the center of the hypersphere. However, it cannot be guaranteed that the neuroimaging data are Gaussian in feature space. The spectral clustering algorithm is very robust to outliers and exhibits favorable performance relative to other graph clustering methods [35,36], but it does not always lead to particularly good solutions [37]. The DEC algorithm, for its superiority in clustering high-dimensional data [12], exists with a relatively low dependence on the internal data distribution structure of each cluster. Thus, in this study, it was conceivable that our DEC-based unsupervised approach achieved higher clustering consistency than both K-means and spectral clustering.

The three parcellated functional subregions were located along the anterior–posterior axis of the hippocampus, demonstrating the obviously different functional connectivity patterns. Our results were consistent with recent studies which examined the within- as and beyond-hippocampus connectivity characteristics, and divided the hippocampus into anterior, middle and posterior parts [17,20,21]. In particular, our results demonstrated that the anterior subregion showed preferential functional connectivity with the anterior default mode network (DMN), including the medial prefrontal cortex and the ACC, while the posterior part showed preferential functional connectivity with the posterior DMN, including the parahippocampal gyrus, PCC and some medial temporal regions. As known, the hippocampus is an important node in the DMN and involved in many cognitive functions, such as memory and spatial navigation [38,39,40]. Our results demonstrated that the hippocampal subregions were distinct in the connections with different default sub-networks, and these differences, in turn, validated the parcellation results. As a matter of fact, several anatomical analyses reported that the anterior and posterior parts of the hippocampus also exhibited significantly distinct patterns of anatomical connectivity [41,42,43], with the anterior part strongly connecting with the medial prefrontal cortex and the posterior part strongly connecting with the PCC. Accordingly, our results may reflect the potential relationship between the functional and anatomical attributes of the hippocampal subregions.

Previous studies reported that long-term navigation experience would change the gray matter volumes of taxi drivers’ hippocampuses, with the posterior hippocampus significantly larger and anterior hippocampus significantly smaller than those of control subjects [23,24]. However, the functional plasticity of hippocampal subregions related to long-term navigation experience remains elusive. Our study investigated the impact of long-term navigation training on the functional connectivity patterns of the hippocampus for the first time. Specifically, in the comparison analysis, the middle and posterior subregions of the hippocampus exhibited significantly reduced functional connectivity with bilateral superior frontal gyrus, left OFC and right ACC in the taxi drivers. These results were in accord with our previous study, which observed the navigation-related reduction in functional connectivity in the entorhinal cortex of taxi drivers [25]. As known, the entorhinal cortex is involved in the conveying of spatial information to the adjacent hippocampus [44,45]. Therefore, the functional connectivity changes in the hippocampal subregions in the present study should be noted. Indeed, when humans navigate, there might be more than one route to the destination. The prefrontal cortex was reported to play a pivotal role in evaluating and selecting the optimal route [46], with the activity of lateral prefrontal cortex shown to correlate with the number of possible paths available at a choice point. The activation of the lateral prefrontal cortex and the superior frontal gyrus was also found when navigators encountered a detour, forcing them to replan an alternative route. Therefore, the reduced functional connectivity of the hippocampal subregions in our study might reflect the altered manner of route planning in the taxi drivers [46]. These results were also supported by the previous studies, which indicated that the hippocampus was not as necessary for navigating in the highly familiar environments [47,48,49]. Specifically, the taxi drivers navigated in the same city for a long time, and their route planning was more likely to be a process of spatial memory retrieving, instead of generating a route temporarily with careful consideration. 

Several potential limitations in this study should be noted. Firstly, the mask of the hippocampus was defined at the group level, which ignored individual differences. In fact, since the volume of hippocampus is relatively small, a group mask for all the subjects may introduce some inevitable errors for each participant. Future studies should focus on a definition of ROI that is more suitable for individuals. Secondly, only 20 taxi drivers and 20 healthy subjects were included in the comparison analysis; the small sample size might limit the statistical power for detecting group differences. Moreover, the relationships between the hippocampal functional connectivity and individual navigational ability were not clarified, since participants’ navigational competence was not quantitatively evaluated. Finally, the comparison analysis was conducted in a specific geographic region; thus, future studies should focus on improving the generalizability of the findings to other populations.

## 5. Conclusions

In this study, we introduced a DEC-based brain parcellation framework for the functional parcellation of the human hippocampus. Compared with traditional stepwise methods, this proposed framework demonstrated higher inter-scan consistency, which indicated its superiority in controlling neural fluctuations within individuals. The bilateral hippocampus was separately parcellated into three functional subregions, and significant differences in functional connectivity patterns between these subregions were observed. Additionally, the comparison analysis demonstrated navigation-related changes of functional connectivity in hippocampal subregions of taxi drivers. In conclusion, this study revealed the functional segregation characteristics of the human hippocampus using the DEC-based framework. The study also investigated the impact of long-term navigation experience on the connectivity patterns of hippocampus for the first time, which might shed new light on the potential influence of extensive navigational training on the functional organization of human brains.

## Figures and Tables

**Table 1:** Demographic information of participants recruited in comparison study.

Variable	Drivers	Non-Drivers	*p* Value
Sample size	20	20	
Age (years)	39.5 ± 5.8	41.1 ± 5.0	0.34 ^a^
Sex (male/female)	20/0	18/2	0.15 ^b^
Education (years)	9.5 ± 1.8	9.0 ± 1.4	0.37 ^a^

^a^ Two-sample *t* test. ^b^ Pearson’s chi-square test.

**Table 2:** Taxi drivers exhibited significantly reduced functional connectivity of hippocampal regions relative to non-drivers.

Target Region		Side	BA	Cluster Size (Voxels)	MNI Coordinates	T-Value
					(*x*, *y*, *z*)	
**HIPP_LM**						
Anterior cingulate gyrus		R	32	19	3, 42, −3	−3.94
Superior frontal gyrus		R	10	16	21, 57, 27	−3.65
Superior frontal gyrus		L	10	11	−15, 60, 27	−3.91
Superior frontal gyrus		R	10	12	6, 51, 33	−3.85
**HIPP_LP**						
Orbitofrontal cortex		L	11	17	−27, 42, −12	−4.96
Superior frontal gyrus		L	10	11	−21, 63, 24	−4.04
Caudate nucleus		L		13	−3, 12, 0	−4.61
**HIPP_RA**						
Superior frontal gyrus		R	10	14	6, 66, 6	−4.63
Inferior parietal lobule		R	40	14	48, −27, 24	−4.32
Superior frontal gyrus		R	10	13	21, 66, 24	−3.93
**HIPP_RM**						
Superior temporal gyrus		L	22	12	−30, 6, −33	−4.70
Anterior cingulate gyrus		R	32	45	3, 39, −3	−5.04
Superior frontal gyrus		R	10	29	6, 66, 6	−4.45
Superior frontal gyrus		L	10	11	−21, 63, 18	−3.82
Superior frontal gyrus		R	10	41	21, 66, 27	−5.35
**HIPP_RP**						
Superior frontal gyrus		L	10	29	−21, 57, 18	−4.01
Middle frontal gyrus		R	10	12	21, 66, 24	−3.99
Caudate nucleus		L		16	−3, 12, 0	−4.45
**HIPP_LA**						
None						

HIPP_LA = left anterior hippocampus; HIPP_LM = left middle hippocampus; HIPP_LP = left posterior hippocampus; HIPP_RA = right anterior hippocampus; HIPP_RM = right middle hippocampus; HIPP_RP = right posterior hippocampus.

## Data Availability

The local datasets analyzed during the current study are not publicly available due to patient privacy, but are available from the corresponding author on reasonable request. Public challenge datasets can be found at: https://www.humanconnectome.org/, accessed on 1 May 2020.
